# The iconographic brain. A critical philosophical inquiry into (the resistance of) the image

**DOI:** 10.3389/fnhum.2014.00300

**Published:** 2014-05-15

**Authors:** Jan De Vos

**Affiliations:** Department of Philosophy and Moral Science, Centre for Critical Philosophy, University of GhentGhent, Belgium

**Keywords:** image culture, virtual, iconology, neurologization, psychologization

## Abstract

The brain image plays a central role in contemporary image culture and, in turn, (co)constructs contemporary forms of subjectivity. The central aim of this paper is to probe the unmistakably potent interpellative power of brain images by delving into the power of imaging and the power of the image itself. This is not without relevance for the neurosciences, inasmuch as these do not take place in a vacuum; hence the importance of inquiring into the status of the image within scientific culture and science itself. I will mount a critical philosophical investigation of the brain qua image, focusing on the issue of mapping the mental onto the brain and how, in turn, the brain image plays a pivotal role in processes of subjectivation. Hereto, I draw upon Science & Technology Studies, juxtaposed with culture and ideology critique and theories of image culture. The first section sets out from Althusser's concept of interpellation, linking ideology to subjectivity. Doing so allows to spell out the central question of the paper: what could serve as the basis for a critical approach, or, where can a locus of resistance be found? In the second section, drawing predominantly on Baudrillard, I delve into the dimension of virtuality as this is opened up by brain image culture. This leads to the question of whether the digital brain must be opposed to old analog psychology: is it the psyche which resists? This issue is taken up in the third section which, ultimately, concludes that the psychological is not the requisite locus of resistance. The fourth section proceeds to delineate how the brain image is constructed from what I call the data-gaze (the claim that brain data are always already visual). In the final section, I discuss how an engagement with theories of iconology affords a critical understanding of the interpellative force of the brain image, which culminates in the somewhat unexpected claim that the sought after resistance lies in the very status of the image itself.

## Introduction

The brain plays a central role in contemporary image culture: it has become an ubiquitous and undeniably influential icon, with its color variations reminiscent of Andy Warhol's Marilyn Monroe prints (De Vos, 2013b). Perhaps the most significant aspect of this is the now prevalent notion that, as the sociologists Nikolas Rose and Joelle M. Abi-Rached put it, the technologies of visualization finally and objectively reveal the physical basis of human mental life within patterns of activity in the living brain (Rose and Abi-Rached, 2013)^1^. That is, the connection of the psychological to the brain would ostensibly appear to pass through the image. For some, this visual link is to be understood quite literally: science writer Rita Carter contends in *Mapping The Mind*, for example, that the brain of a person driven by obsession is “frenzied” while a “depressed brain” shows a dull glow (Carter and Frith, 1998).

The primary aim of this paper is to probe the powerful interpellative allure of these brain images, as they are colored by psychology. The eagerness of the press in this visual-centric time and age, where the image (especially the digital image) is a privileged commodity, is one thing, but the other more pertinent question is, what is the status of the image within wider culture, and more specifically, within scientific culture and science itself? For even though brain imaging is not the sole method within brain science, it most certainly takes a central place -just consider the exponential rise of fMRI studies since 1990 up until now (Logothetis, 2008; Rose and Abi-Rached, 2013). Hence, if the philosopher and cognitive scientist Fodor (1999) is right to argue against oversimplifying localization research and to question why we are spending so much time and money on it, then to truly understand this requires delving deeper into the power of imaging and into the power of the image itself.

Having said this, the paper thus represents a critical philosophical scrutinizing of brain imaging, of the brain *qua image*, focusing principally on the issue of mapping the mental onto the brain image and explicating how, from there, the brain image functions within contemporary culture and why it has come to play a pivotal role in processes of subjectivation. Hereto, I will draw upon Science and Technology Studies juxtaposed with culture and ideology critique and different theories of image culture.

The first section relates the omnipresent brain image to subjectivity and ideology setting out from Louis Althusser's concept of interpellation and his conceptualization of subjectivity. This will allow me to tease out the central critical philosophical question of this paper: what could serve as the basis for a critical approach, or phrased otherwise, where can a locus of resistance be found?^2^ In the second section, drawing predominantly on Jean Baudrillard, I will focus more closely on the dimension of virtuality, specifically as this is opened up by what I call *brain image culture.* This leads to the question of whether the digital brain can be opposed to old analog psychology: is it the psyche which resists? This will be taken up in the third section where, however, it will be concluded that the psychological cannot be the locus of resistance sought for. The fourth section, then, explores how the brain is constructed as an icon, and more specifically, from which gaze this emanates. In the fifth and final section, an engagement with theories of iconology will afford a critical understanding of the interpellative force of the brain image, and will allow me to claim, somewhat unexpectedly perhaps, that the requisite resistance lies in the status of the image itself.

## The brain image as an interpellation of subjectivity

The omnipresence of the brain image in contemporary culture cannot but have effects on how we see ourselves and society (Hacking, 1998; Slaby, 2010). Indeed, the anthropologist Joseph Dumit argues that the brains we encounter in magazines and newspapers, on television, in a doctor's office, or in a scientific journal “make claims on us.” Louis Althusser's perniciousness concept of hailing might be useful here: one could say we are interpellated by the brain images. We are called upon to answer them, to *subjectivize* ourselves in relation to them. This is the Althusserian “surplus in the recognition”: interpellation, as an ideological operation, produces a subject. In the case of the brain image, then, its message, *look this is what you are*, can be said to engender a subject: *oh, really, is this me?* Or as Dumit puts it:

As people with, obviously, one *or* another kind of brain, we are placed among the categories that the set of images offers. To which category do I belong? What brain type do I have? Or more nervously: Am I normal? (Dumit, 2004).

One can already discern here a question so obvious that it risks being overlooked: can the neurosciences account for the *surpluses* it invokes qua subjectivity? That is, if the brain sciences, and, more particular yet, those involved in brain imaging, are engaged in both researching subjectivity and producing subjectivity, then how to account for the multifarious short-circuits and circularities that this entails?

Of course, one could immediately object that neuroscience does not necessarily deal with subjectivity *per se*; rather, its jurisdiction could be said to be limited to investigating the general principles of the neural system. And by way of a second objection, one could also say that not all neuroscience research results in or aims at the production of brain imaging. However, would it not be fair to argue that even the most basic of neurological research cannot but, in one respect or another, impinge upon the dimensions of subjectivity and the psychological? Indeed, when all is said and done, the locus or terminus of neural tissue is the brain, and the latter is, arguably, the very organ of subjectivity, regardless of how the latter is conceptualized. Secondly, does basic neurological research not also invariably produce scientific data, which is then subsequently used in brain imaging technologies and its logarithms? At a bare minimum, a sober questioning of the function of the image within the brain sciences and the indisputable weight it carries within the field of neuropsychology might be of some value to those branches not directly involved with the psy-factor or with imaging as such.

Ultimately, if the brain image has a saliency within contemporary culture, then it is clear that it will also have surplus effects in scientific culture and in science as such. In this respect, Beaulieu (2002) notes that the argument that pretty pictures above all serve popularizations does not account for the ways in which these representations also pervade research environments. The latter can hardly be understood as taking place in a vacuum; or as Cohn (2008) has put it, research environments are not a *non-space*.

Many authors, in this respect, have pinpointed how political, economic and cultural factors shape even the most basic neurological research (Slaby, 2010). Even at the level of primary neurological research, then, there are ideological surpluses to be discerned: neuroscientific research is *prima facie* colored by politics, economics and culture, which, in turn, colors -if one will permit this use of imagery- the resulting brain image which is produced. Is it not precisely these surpluses which subsequently hail the lay person and engender the Althusserian “surplus in the recognition?” We are not merely interpellated by science in other words, but also by the ideological and cultural germs these sciences, and the images they produce, host.

However, the important question then becomes whether the interpellative process on the behalf of the brain image can be fully apprehended within a strictly Althusserian framework of *imaginary misrecognition*? Although the ubiquitous brain imagery does undeniably present us with a glaring, unified and Gestalt-like image to identify with, one can no longer frame this, à la Althusser, as offering the illusory promise of being an autonomous subject. In fact, is not the message the brain images convey, rather, the exact opposite: *look, you are nothing but this automaton*? Patricia Churchland, for example, in spite of the fact that she rejects the “neurojunk” of “free choice/self is an illusion,” argues that making decisions, going to sleep, getting angry, being fearful… are just functions of the physical brain (Churchland, 2013b)^3^. At the least, then, the interpellation of brain science seems far removed from an Althusserian conception of it as rendering people unfree by endowing them with an illusionary sense of freedom, agency and causality. Rather, brain science and its images actually deconstruct these categories and, most importantly, deconstruct the subject itself: you are not even unified, but, rather, as it were sliced up by the brain image and dispersed in the neural network. Let me be clear, the issue is not to disparage this aforementioned deconstruction, nor to attempt to resurrect some kind of unified or agential *extra-neural* subject; rather, I am concerned with understanding the unexpected intricacies of the interpellation of brain images.

The key issue lies in discerning how the message carried by the brain image, “look, this is what you actually are,” once it has permeated popular culture, not only invites us to identify with the icon, but also invites us to adopt the iconography. That is, our self-understanding and self-consciousness is solicited by both the images and the signifiers stemming from discourses on the brain. It is important to grasp that what one is actually being called upon to identify with here is not the brain image as such, the paradoxical Gestalt signaling the end of unity and agency, but, rather, the perspective of neuroscience itself. We are, as it were, being hailed into the position of the neuroscientist observing the brain. Or, phrased otherwise, where there are images it is crucial to discern the operation of the gaze, and the point from where the latter departs. Hence, if brain imagery does indeed bring into being a *subject*, this is very specifically an academic subject: inasmuch as brain images and neurological signifiers structure our self-understanding *from the academic vantage point* we are called upon to adopt.

On the one hand, this remains consistent with an Althusserian conception of the subject as effect and function of a discourse and of a (power) relation. But, on the other hand, it differs significantly from the Althusserian subject who is called upon to identify with its designated place: for rather than effecting an identification with the image itself, the brain image actually calls upon us to identify with the point from where the image is constructed.

This can be connected to older forms of psychologisation (I will discuss the issue of psychology and psychologisation in great detail in this paper): just as the dissemination of psychology throughout society transformed each of us into our own psychologist (De Vos, 2012), now we are called upon to become the neuroscientist of ourselves. Today, the omnipresent brain imagery not only directs the gaze inwards, but, more importantly, imposes upon us the neuroscientific vantage point from whence to launch that gaze. This allows me to formulate an altogether different critique from the well-known Foucaultian strands that argue that (popular) neuroscience discourses encourage particular kinds of selves who are then more or less amenable to certain political agendas (see e.g., Rose, 2006; Johnson, 2008). By way of contrast, I argue that via the process of interpellation, the subject shifts position from the neuro(psychological) object he or she is said to be, toward the external observatory position: fascinated by the brain scans it exclaims, *look, this is what I am!* This is how we can begin to fully apprehend Althusser's “surplus in recognition,” as that aspect which assumes the neutral and sovereign position of Academia. And it is precisely here, in that ostensibly neutral and naturalizing scientific gaze, that one could claim that ideology comes into play: that is, it is in the alleged neutral, objective, and natural portrayal of mankind—here I follow Slavoj ŽiŽek^4^ —that one finds ideology at its purest. Our identification with the ostensibly neutral and a-political vantage point of Academia is the paradigmatic form of ideological interpellation today.

But we should not stop here, for is this position not a *virtual* position by virtue of the fact that it places us in a transcendental, non-existent vantage point? One encounters here a remarkable peculiarity overlooked by neuroscience and perhaps also in its critiques: it is only a small step from materiality to virtuality. Unquestionably, despite its claim to be the materialist approach par excellence, the neurosciences increasingly find themselves, albeit for the most part unknowingly, in the virtual dimension. Consider the notion that the mind is but the software of the brain and might 1 day be uploaded to a computer^5^. Although these fantasies have often been contested, at the very least they demonstrate how the neuroscientific approach most readily solicits the virtual. A number of critics have also touched upon this theme, such as the anthropologist Allan Young who argues that we have entered the era of “Human Nature 2.0.” (Young, 2011), or Jan Slaby who noted that the concept of mirror neurons has come to function “as a neural Wi-Fi that links us up to form various social networks” (Slaby, 2013a). However, to the best of my knowledge, no extensive study has yet been carried out exploring why virtuality and the neurosciences so readily invoke each other.

In this regard, it is noteworthy that the European Union launched a huge research program entitled “The Human Brain project, HBP” the aim of which was to design a super computer which could provide us with an *in silico brain*. The goal of the project is to build a new information computing technology infrastructure capable of integrating all the available data on the brain, in order to arrive at “detailed computer reconstructed models and simulations of the brain”^6^. As said in a recent promotional video:

Through this new in silico neuroscience, there will be nothing we cannot measure, no aspect of the model we cannot manipulate, there will be no question we cannot ask^7^.

However, the true problem of this unabashed ambition to “gain fundamental insights into what is means to be human” (Walker, 2012) might not be that it would lead to *in silico* knowledge losing sight of the real, concrete, and embodied human being. The real issue, rather, might be that it fails to understand that it deals with an always already virtualized subject. Just consider how, by virtue of the interpellative procedures described above, contemporary subjectivity is always already necessarily marked by the scientific imagery, and entails a subject that shifted position to the virtual academic vantage point from where it contemplates its avatar, that is, the neuropsychological object it is said to be. In a similar, and not altogether unrelated, way, a neuroscience in the grip of the image and virtuality might fail to grasp that today's subjectivity always already passes through image culture and virtuality, as these increasingly define our life-world and personhood (just think of social media, apps and other rapidly evolving ICT-applications). The project, then, runs the risk of getting lost in the mirages of the virtual, as it mistakenly supposes that it is modeling a pure fleshy, material subject.

Hence, a further aim of this paper is to disentangle some of the short-circuits and circularities in today's neuroscientific image culture. If, as a consequence of these circularities, brain imaging is, indeed, at risk from getting lost in virtuality—as exemplified in the unquestioned assumptions of “The HBP”—the crucial question appears to be how one can resist the compelling interpellative force of such brain images, seemingly all the more powerful in their *digitality* and virtuality? But, as will become clearer, this is not the right question after all, for if subjectivity is in fact something which cannot be cut loose from the reigning and hegemonic icons and iconographies of its times, then it would be a mistake to look for a subject *beyond* the image. In such a scenario, and in line with the philosophical tradition of Immanuel Kant that combines subjectivity with critique (*sapere aude* positions the modern subject as a critical subject), the question of what might form the basis of a viable critique of the hegemony of neuroscientific virtualized image culture, becomes an altogether different one. If, for Kant, the modern human being was to use his/her own reason (as opposed to complying with tradition and power), the fundamental issue became a kind of reflective critique: that is, the discerning of the conditions and boundaries of thinking itself (Kant, 2005). In relation to brain imaging, then, the central question becomes: what are the conditions and boundaries of imaging itself? Or, said differently, what resists it? Hence, resistance here is not in the first instance conceived as opposition to the alleged deterministic or reductive implications of the technological gaze^8^ - e.g., the negative *psychological* effects of neuroimaging- but, rather, as that which delimits (neuro)imaging from within. This resistance, the paper will argue, and somewhat unexpectedly perhaps, derives from the image itself. It is only from there, I will subsequently claim, that the actual deterministic or reductive potentials of the technological gaze (and their ideological) bearings can be discerned and eventually criticized.

But, in the interim, there is considerable preliminary work to be done: the first task is to inquire further into the close connection between brain image culture and virtuality, which will involve drawing extensively on the French theorist Jean Baudrillard.

## “the spectacle of the brain” and virtuality

All that fascinates us is the spectacle of the brain and its workings. What we are wanting here is to see our thoughts unfolding before us - and this itself is a superstition (Baudrillard, 1988).

We want to see ourselves, we are fascinated by the made visible brain, that thing that does all that psychological stuff of thinking, wanting and desiring. Perhaps this is why we denounce the idea of rational agency, free will and love altogether. Because when we observe ourselves, via the image of the brain, we take a position outside or beyond cognition, will and desire, and from this place the latter appear as nothing other than mere chimeras. The Althusserian surplus in recognition is, as such, that precise point beyond our own psychology: the spectacle of the brain engenders the spectator, a paradoxical and emptied out agency outside of itself.

However, as my earlier reference to psychologisation expressly indicated, this particular organization of the gaze was already in place within old-fashioned, pre-neuroscientific psychology. Indeed, the latter, from its very infancy in the scientific age, attempted to establish itself as a technology for the visualization of the human—just consider the one-way mirror, the Gesell Dome or, for that matter, the use of hidden cameras. These technologies amount to the construction of an external gaze from where one is allegedly able to see the true face of human beings. Even the use of statistics in the psy-sciences can be understood in terms of the same visual register: the numeric data in the end contributes to the visualization of human behavior within charts and graphics. It is from within the dominance of this visual register that the so-called lay person is addressed: *look, this is what you are*. Hence, through what can be called processes of psychologisation—which form an inextricable part of psychology itself (De Vos, 2012)—modern man is interpellated to adopt the external gaze and look upon the *homo psychologicus* he or she is said to be. Such a procedure is repeated through the neuroscientific interpellation: you are presented with an image of your alleged final ground and this redoubles you in, on the one hand, your brain avatar and, on the other hand, a more obscure and not always acknowledged position (an additional *you*) from where you contemplate yourself.

Of course, one can take a step back further still from modernity and the advent of the sciences and argue, with Jacques Derrida, that the human being dwelling in Logos is always already “tele” from itself (Derrida et al., 2002): this concerns the fact that as a speaking being the human being is separated (epistemologically and ontologically) from itself. Or as Jacques-Alain Miller puts it, the mere fact that one speaks always already transforms “what is” into a fiction (Miller, 2002). It is at this precise point, that where Logos allows the human to make an abstraction of what he or she experiences, that the importance of images comes into play. Language allows the *envisioning* of the world, of others and oneself. In Logos there is always the gaze and the other scene, and it is in this sense, moreover, that human existence, as a cultural, discursive, and social issue, arguably has always encroached in one way or another into the domain of virtuality. Suffice to think of the historical (religious or other) constructions of an imaginary space or time, entailing either a pre-world, a beyond-world or a parallel world (e.g., the Greek mythology, the Christian concept of paradise, the colonial image of the Americas).

However—to amend this diachronic meta-perspective with a synchronic one—in modernity this scheme can be said to receive a very specific turn of the screw. With Immanuel Kant denouncing access to “das Ding an sich” as foreclosed, the image was bound to take up a more central role than ever before. Modern science, and with it modern man, thus renounced all claims to have unmediated access to reality or being. It is here that the reign of the image truly begins. For, as the Flemish philosopher Marc De Kesel argues, in modernity the image loses its pre-modern grounding and its connection with reality: it is no longer real or natural, and hence, newly unbound, starts to proliferate in an unseen way (De Kesel, 2007). The seemingly unstoppable multiplication of images has to ward off the lack of a firm ground in ontology. It becomes clear that modern image culture does not operate as a form of mediation between us and the real, but, rather, engages in a frenzied process of constituting a virtual space and reality on top of the gaping ontological abyss. It is in this respect that De Kesel argues, given that modernity signaled the end of any claims to a direct connection to being, we have henceforth become addicted to images: “we only exist insofar if we succeed in imaging ourselves” (De Kesel, 2007).

It is at this specific point that the paradox of brain imaging comes in: brain images are believed to show us how real, natural and organic we are, how we are all made of flesh and blood. However, it can also be argued, given their status as digital and virtual constructs, rather than mediating between us and our material self, they actually draw us into virtuality. To put it in Baudrillard's terms, they concern the *real more than the real*. Baudrillard uses this phrase to describe the obsession with the real so integral to the mythology of our ultra-mediatized society. It is this voracious demand for reality, truth and objectivity that he sees at work in live reporting, the newsflash, the high-impact photo, the eye-witness report, etc. It is the “truer than true” which counts, or “the fact of being there without being there” (Baudrillard, 1998). Cyberspace, and more generally, virtuality, I claim, is the ultimate locus of this *real more than the real*. To illustrate this, let me use an anecdote: I met someone at a social occasion, and after having spoken about what each of us did for a living, she subsequently asked how my name is spelled so that she could google me online. To use Sherry Turkle's quip: instead of taking me at face value she wanted to take me at my “interface value” (Turkle, 1995). In other words, today, the *real more than the real* resides in the digital sphere, within cyberspace. For example, as we are all now well aware after Edward Snowden's revelations, knowing what people or organizations really think or are up to requires that you skim and hack digital networks. This *real more than the real*, as the very definition of the virtual, is also a central element of neuroscientific imagery. The crux of the neuroscientific findings as they are crystallized in the brain image is that, as Baudrillard puts it, “I was not there”: the brain imagery essentially poses the paradox of “being there without being there.” The brain has its own reality, a reality which we ourselves have no part in, where we are not present. This is especially tangible in Robert Nozick's well-known thought experiment, in which he asks whether we would be willing to plug our brain into a supercomputer that would provide us with any experience we desire (Nozick, 1974). Intended as an argument against philosophical hedonism, Nozick's experiment above all shows how the brain, precisely by pushing to virtuality, cannot but pose the question of “being there” in all its paradoxes. The brain confronts us with a truth and a reality from which we, potentially at least, are ourselves absent.

Now, if this issue is more often than not acknowledged by neuroscience itself, the question nevertheless still remains: is the recourse to the brain sciences wholly adequate for our attempts to assess this paradox? For Patricia Churchland, for example, seemingly it is. With little or no fuss she observes that one's love for one's child is simply a matter of neural chemistry, although, she does acknowledge, “[c]oming to terms with the neural basis of who we are can be very unnerving” (Churchland, 2013a). How should we understand Churchland's seemingly casual use of the notion of “unnerving?” Is she inferring that even the deconstruction of our self can be accounted for in neural terms? Or does it imply a more unsettling conclusion: the brain un-nerves us, that in the end it de-brains us. Even if one could dismiss this as mere metaphorical hair-splitting or an exercise in pedantry, the question nevertheless remains: can the absence of agency, or of subjectivity as such, and the resultant uncanniness this provokes within a “subject” really be accounted for by the neurosciences themselves, or must we take recourse to the old psychological models in order to elucidate the “psychological” effects of brain imaging?

Am I not stumbling upon here the horizon of brain imaging, or the point of resistance I was searching for? That is, it is the psychological, so it would seem, that might be the critical component of the brain sciences and its production of images. In order to consider this possibility, and, ultimately reject it, in the next section I will more substantively question the recent shift from psychological models to neurological models, a shift which seemingly runs parallel with the shift from analog image culture to digital and virtual culture. I will explore this through recourse to Jean-François Fogel and Bruno Patino's claim that digitalization does have its frontier, in addition to drawing upon relevant art history theories pertaining to the image and the photograph.

## From the psychological portrait to the disembodied brain image

It is tempting to propose that the psychological will never dissolve without remainder within the neurological. Consider, for example, the argument that brain imaging techniques only envision coarse psychological traits and that, as such, the ephemeral reminiscing that passes through the mind like a soft breeze, the subtle gesture or the intricate glance exchanged between two people, will forever elude visualization and digitalization. However, if there is one thing that the rapidly evolving digital imaging technologies have showed us, it is that what is considered to be analog and non-scanable today will be fully computable and chartable tomorrow. Take as a case in point the development of automatic emotion recognition systems which analyze faces (still photos or moving faces captured by a camera) in order to determine emotional states^9^. Consequently, it would be foolhardy to argue against the notion that even a single goose bump will eventually have its correlate in the chemical or electrical status of the nervous system, and thus be, as a result of the continually evolving technology, fully measurable and digitazible. However, the French authors Fogel and Patino proclaim that digitalization does have its frontier:

Any innovation will eventually be exceeded, the only sustainable element is the connection. A login added with a password to access a network: this is the lightweight baggage that everyone is guaranteed to carry tomorrow (Fogel and Patino, 2013, my translation).

Is this the ultimate frontier of digitalization: login and password, the outstripping and mark of subjectivity proper, good-old analog psychology as both condition and exception of the digital and the virtual? Login and password, inasmuch as they allude to a certain intimacy or a secret even, can be said to pertain to old-fashioned psychology; that is, to the psychological *agalma*. Although, of course, passwords were always already not exactly exempt from digitalization, inasmuch as the computer did know them after all. But this could still be considered within a psychological framework: your password was only known by the Big Other (to use the Lacanian term), albeit a technological Other. However, it would appear that the days of the psychological, semi or pseudo-analog password are truly numbered, as today more and more electronic devices are activated with biometric keys, e.g., scanning your iris or your fingerprint. Biometric access seems not only more secure but also more idiosyncratic than the analog-psychological password. The ultimate step here will be for the digital to connect, not to the potentially digitizable flesh, but, rather, to the digital of the human body. That is, the ultimate biometric access would appear to be our genetic code: *please lick here*! Or, alternatively, the digital network directly connected to the digital of the human^10^.

At the very least, the conclusion to be drawn here is that your unicity is not psychological, which is to say also that it is not this which resists virtualization and visualization, and, for that matter, neurologization. The digital brain image is thus the looking glass through which psychological categories (such as free will, love, empathy, etc.), become neuroscientific issues. The psychological is in this way gradually emptied, becoming, on the contrary, bio-neurological and, in turn, (potentially) fully scanable and digitizable, if not wholly digital as such. Hence, as it becomes indisputable that thinking, willing, desiring, or even Marcel Proust's madeleine-experience for that matter, all depend on things going on somewhere in the brain, then it would appear to make little sense to hold on to a psychology which is rapidly melting away.

It would seem, ultimately, that we have to agree with the presumptuous ambitions of the HBP: when modeled, the brain becomes fully malleable and answers to any question. With digital brain imaging, then, we have made ourselves visible, and hence, seemingly, fully accessible. In psychological imagery the human subject had its dark side, the spoken pointed to the unspoken, the thought to an un-thought, consciousness to an unconsciousness. The digital brain image, in contrast, allows potentially full access: just move the cursor, zoom in or zoom out, set the angle, adjust the parameters, change the colors… The Harvard Medical School, for example, offers a free online MRI atlas which simultaneously shows horizontal, sagittal, and coronal sections through which one can navigate, whilst displaying any level of the hemispheres, brainstem, and even some spinal cord, all the while using different MRI weighting (T1 or T2) and PET too^11^. In principle, then, there is no blind spot, no inaccessible area; at the most there is a *sub-consciousness* which, in turn, can be made visible.

In this way, the brain image is not a psychological portrait. Portraits have traditionally been viewed as revealing some insight about the figure represented in the portrait (Ayers, 2011) and opening up some interior space (Pearl, 2010). For Drew Ayers, photographs, while depicting embodiment, potentially uncover the truth of things, so that they—and it is here that he leans on Roland Barthes and André Bazin- expose “the inner workings of an object or person” (Ayers, 2011). Similarly, Shawn Michelle Smith contends that the portrait was believed to be able “to depict the inner soul of an individual in a representation of external countenance” (Smith, 1999). Hence, the portrait, belonging as it does to the realm of the imaginary, not only points to the real of the body, but also functions as, to use Ayers' terms, “both [the] index and icon” (Ayers, 2011) of the inner person depicted within the image. Simply put, the truth-value of the portrait concerned the psychological: the soul.

This is not at all the case with brain imaging: it does not begin with the depiction of a supposed embodiment, which then serves as the index of the terra incognita of the psyche as the supposed core of the human. Rather, brain scans show and lay bare, more or less accurately and probably more and more conclusively in the near future, (the very base of) the psyche, or at least that which has previously been referred to by that designation. Sigmund Freud's “andere Schauplatz” has thus been tracked down and lost its independent status. Or, as Churchland has it: “I am who I am because my brain is what it is” (Churchland, 2013b). In this way the brain image is not a portrait, a representation that points to something else; rather, it is a pure self-reflective image, a pure index or icon of the Real itself.

In the end, then, it is not only psychology that is evacuated, but also the body. Ayers sees this at work in the so-called DNA portrait (whereby commercial services offer “personal DNA pictures” based on the analysis of a sample of your DNA) in which, he argues, the body as surface is lost (Ayers, 2011). Precisely the same form of dis-embodiment, I contend, is at work in the brain image as it proliferates in wider culture. After all, is the paradigmatic brain image within the everyday public sphere not that of the singled-out brain, presented to us as faceless, sexless, classless, raceless, and, ultimately, bodiless? Just consider Daniel Amen's online SPECT gallery,^12^ which depicts a panoply of more or less colorful schematized brains. Or consider how governmental and other campaigns connected with brain research, whether in terms of their academic or non-academic communication most often use logos figuring isolated brains^13^. But perhaps the most salient example of these segregated brains is the increasingly popular images of the *connectome*: as most of them lack the contours of even the skull or of the brain cortex itself,^14^ they seem the ultimate trope of the bodiless stand-alone brain. Even in (f)MRI brain scans, in the particularly rare instances in which we see a nose, lips and especially eyes, does it not all too quickly become uncanny if not utterly obscene? At the least, and contra Casini (2011), one could argue that brain images are not portraits as they do not look back—the latter property, according to Nancy (2006), being characteristic of late modern portrait photography.

The paradox is that when shown one's own photograph, one often finds it difficult to identify with, whereas the digital brain image appears far less problematic to relate with, as it is supposed to represent you at your most natural. As BBC-journalist Evan Davis puts it after having an MRI scan of his brain:

I'm just fascinated by the pictures (…) That's my brain? That's my head…. That's quite a good picture isn't, you could recognize it as me (my transcription).^15^

But, precisely because you had no access to your psychological-analog core, your psychology, in turn, had an intuitive weight and presence that you could claim. Access to your bio-neurological unicity, contrastingly, is wholly possible and relatively unproblematic as it is mediated by the digital brain image, but it reveals to us that any intuitive subjective weight and presence are merely illusory, or better yet, virtual. Or in the words of the artist Susan Aldworth, “You can look INTO my brain but you will never find me”: the brain image signals a non-presence or perhaps even the weightlessness of the psychological: “I am both in my head, and out of my brain” (Aldworth, 2011). Even if Aldworth, at first glance, appears to look for a Self beyond the brain scan, she can eventually be said to situate this surplus outside the psychological realm: while undergoing a cerebral angiogram (for medical reasons) she relates how she herself watched the produced images on a computer screen:

Looking up at the screens, I could see the inside of my brain with my eyes - my brain was working, while I was looking inside it. I will never make sense of that moment (Janes, 2000).

This testifies to the fact that the compelling interpellative power of the brain image concerns not so much the injunction to identify with the brain as such, but, as noted prior, to identify with the academic gaze. Is the conclusion, then, not that there is still in fact an “andere Schauplatz” after all? If this is indeed the case, then this warrants a closer scrutiny of the structure and the dynamics of the gaze, starting out from that sovereign place and then delineating the ways in which the brain is subsequently constructed as a particular powerful and central icon within contemporary culture.

## The iconographic brain and the data-gaze

But, firstly, if I refer to the brain as iconographic, then what exactly is an icon? As Susan Buck-Morss explains, the icon should be understood in its Christian-Platonic lineage: that is, it is precisely at that point where the relation between ideal forms and earthly forms presents us with the enigma of their connection, that the icon comes in. Buck-Morss' central example is that of sovereignty: “The sovereign is an icon in the theological sense. He (or she) embodies an enigma—precisely the power of the collective to constitute itself” (Buck-Morss, 2007). This enigma can be comprehended as follows: constituted power cannot but be its own ground, it has to be its own constituting power. Or, to put this in terms of the law, the law, as it founds a community, necessarily has to be called into life (and sustained within its life) from a position before (and beyond) the law. The closing of this circle, Buck-Morss contends, demands a miracle: “and the icon of the sovereign figure provides it” (Buck-Morss, 2007). The icon thus can be said to effect a short-circuit; an impossible but effective closure of sovereignty and the law whereby they paradoxically constitute the very ground on which they are standing upon.

Is not the same issue at stake in relation to subjectivity? For must the subject not also assume its own subjectivity, whilst lacking the very grounds on which to do so? Especially since the advent of modernity, from that moment when God was no longer able to provide indisputably the final ontological guarantees, the subject must be both the constituted and the constituting subject. Hence, the subject too requires an iconic relay between its ideal and earthly form. This function today, I suggest, is no longer fulfilled by the abstract icon of the Soul, but by the concrete brain image. Observing its dominance within contemporary popular culture, it is evident that the brain has become today's predominant icon, serving as the impossible bridge that traverses the ontological abyss on which the human being founds itself. The brain image makes visible, embodies and fleshes out the essence of the human being, thus short-circuiting the enigma of subjectivity. But, of course, if the specificity of the icon is such that it performs its function by transposing an impossibility into the register of the image and the visible, the question then becomes which gaze in particular is mobilized?

Within the Christian-Platonic iconography it is the transcendent gaze of the figure of God that is at play. Recall Walter Benjamin's argument that in Homer's time the human was above all an object for the gaze of the Olympic Gods (Benjamin, 2008). Similarly, in this respect, ŽiŽek refers to the gigantic Aztecan figures of animals and humans that could only be seen from a view-point far up in in the sky (ŽiŽek, 2002). The gaze that an icon mobilizes thus goes back to the gaze of a transcendent instance. Buck-Morss, in this regard, demonstrates that the roots of today's modern “empire of the gaze” (as she refers to the “global media industry”) can be traced back within the history of Christianit^16^. By way of an aside, perhaps this goes some way to explaining why a number of authors have discerned a religious component to neuroscientific imaging. Slaby, for example, points to the ritualistic and quasi-religious connotations of the fMRI-procedure, in which the operators take on the role of priest-esque figures (Slaby, 2013b). In this respect, it is clear that the central ambiguity of Christian iconography, that is, of a non-material God becoming flesh, does indeed return in cerebral iconography: after all, the brain image does show us both a material and a non-material, virtualized human subject. The only difference today, is that we have substituted the omniscient eye of God with that of Science. As argued, the brain image very specifically interpellates us into adopting an academic point of view from which to look back upon ourselves, others and the world itself. Since modernity, then, the human is both the subject and object of and for the sciences and their Archimedean Gaze.

But, of course, immediately one then raises the question, how does science, and more specifically brain science, construct this gaze? In this regard, Amit Prasad perspicaciously argues that, although the new medical imaging techniques share some similarities with “nondigital visuality,” they do not involve “seeing” in the traditional sense: MRI, for example, is not based on the reflection or absorption of light or other electromagnetic waves (Prasad, 2005). Rather, the newer imaging techniques rely on measurements and computations. Hence, as Beaulieu points out, neuroscientists concerned with basic, non-clinical research (e.g., functional imaging) often rail against the reduction of neuroimaging to its pictorial component. They claim to do quantitative and experimental work, as opposed to visual and observational work. Concerning the images they produce, they argue: “They're not pictures, they're statistical maps” (Beaulieu, 2002).

The common answer to the question concerning why one goes from measurements and data to pictures is that visualizations are used because of the complexity of the quantitative data. As Beaulieu writes: neuroscientists argue that in the end vision is the sensory modality with the broadest bandwidth (Beaulieu, 2002). Following from this, one could go onto argue that, by making the computer's datasets accessible to the human being through the medium of the image, the contingencies come in. But are we really starting off with non-visual data? Is the visual, therefore, only a necessary detour in light of the fallibility of human understanding and its preference for the pictorial? If that would be the case, then one might seek to avoid the image as much as possible; would not, for example, in the future, a full computerized diagnosis of a tumor no longer require the detour to the visual realm and the clinician's gaze?

However, a closer examination of the rationale of the different brain imaging technologies might suggest that the primordial data are always already affected by the visual register. Just consider how the basic premise of imaging technology is the consideration of the brain as a three-dimensional, spatial object. Measuring, then, is a matter of, as Beaulieu describes this in relation to PET technology, establishing a relation between the space of the brain inside the scanner and the space of the digital image. That said, Beaulieu contends that, notwithstanding the reluctance or ambiguousness vis-à-vis the visual, much of the empirics of brain imaging is achieved by using pictorial conventions to render space (Beaulieu, 2002). Taking this argument one step further, I'd argue that, given that this data is de facto spatial, then they are also always already embedded in the visual register. It is therefore not a question of a secondary translation of non-visual or non-pictorial data. Rather, as Beaulieu drawing on Andreasen et al. (1992) already noted, in brain imaging technology the observer is technically incorporated into the machine (Beaulieu, 2002). In other words, the measurements are made, the data is gathered by teleporting, as it were, the gaze of the observer into the scanner.

Hence, as it has already been argued that for the computer to generate the data, a substantial amount of editing has to be carried out (see for example Ortega and Vidal, 2007; Slaby, 2013b), it is my contention that this editing starts precisely from the incorporation of a subjective perspective within the technology. That is, the scanner's vantage point, I claim, is akin to the way a human eye would peer into a sliced up body part. Consider also how, as Prasad remarks, the noise and the inconclusive data have to be filtered out with the help of the so-called reference or body atlases comprising ideal or normal types of human cerebral anatomy (Prasad, 2005). This, in my opinion, shows that the production of data is anthropomorphized, as it were. The especially versatile gaze of the scanner, going from axial, to sagittal to coronal (with further differential viewing based on the differences in relaxation times of hydrogen atoms after magnetization, or in terms of proton density), is in the end constructed as if a human subject were the carrier (or in the appropriate Latin term, the *subjectum*) of the gaze. In short: the data-gaze of the computer is modeled upon the model and the abstraction of the (super)human gaze.

It is precisely this, of course, that complicates the process of interpellation I described earlier, in which the brain image evokes a subjective surplus within the process of recognition. At the least, it is notable that the idea that, as brain images become popularized, the folk psychological gaze of the layman easily goes astray and needs guidance from the expert (who has the appropriate way of seeing the image as condensed and rendered data) is deeply flawed in two respects. Firstly, because the alleged pristine data is always already visual inasmuch as they are the result of the scientific gaze; and secondly, because one risks skipping over the fact that it is precisely with this gaze that the modern subject identifies him or herself via the process of interpellation. That is, the alleged spontaneous, folk psychological gaze of the layperson (easily derided by the senses and seduced by the image) is but a mere fiction. If the layperson is in fact fooled by images, then it is precisely in his position as a proto-academic subject.

To this scheme, just one more twist is needed which is provided by Prasad's argument that the new medical imaging techniques represent a “cyborg visuality” (Prasad, 2005). In other words, it is precisely through the intricate connection of data with the visual in brain imaging that the virtual comes in. Consider Prasad's point, reached through recourse to the work of Anne Balsamo, that in imaging technologies the human body seems to have lost its materiality and instead becomes a visual medium (Prasad, 2005). It might be appropriate to suggest that, by virtue of the technological data-gaze the human turns into an immaterial dataset and becomes its own avatar. Hence, in contradistinction to one of the neuroscientists cited by Beaulieu, who in his rejection of the visual contends: “So once the field grows up [and] becomes less interested in mapping, it will be numbers” (Beaulieu, 2002), my argument is, rather, it will be virtual.

However, within this complex assemblage one crucial issue remains: what is the status, then, of *the brain qua image*? Does it still have a meaning of its own, and if not (as it is merely one of the points within the schema of the gaze), how are we then to understand its interpellative force? These questions concern the iconology of the brain.

## Toward an iconology of the brain image

Iconology, following the seminal understanding of Erwin Panofsky (1972), differs from iconography: while iconography is descriptive, the linking of artistic motifs with themes, concepts or conventional meaning, iconology concerns the interpretation of the images' meanings. That is, as Panofsky writes, iconology “is apprehended by ascertaining those underlying principles which reveal the basic attitude of a nation, a period, a class, a religious or philosophical persuasion—qualified by one personality and condensed into one work” (Panofsky, 1972). For Panofsky, this level of “intrinsic meaning” was to be understood as somehow beyond the conscious volition of the individual artist. However, for my attempts here to construct an iconology of the brain image, William J.T. Mitchell's recalibration of Panofsky's ideas is crucial. Mitchell can be said to have substituted the issue of the basic attitudes of a nation, period, etc., with the question of what the image itself wants (see Mitchell, 2005). That is, for Mitchell, iconology does not concern the interpretation of the images' meanings, but the interpretation of the images' desires. The seminal question is thus:“what do pictures want?” (Mitchell, 2005). Arguing against the dominant perspectives that approach visual culture interpretively and rhetorically, Mitchell wanted to assess what pictures mean and do, and account for the transfixing power they possess on their own terms. For Mitchell, images are not merely inert objects conveying meaning, but, rather, they are like living organisms, things that have desires, needs, appetites, demands, and drives all of their own.

From here, the question can be asked: what does the brain and/or the brain image want? Or, put differently, what is the lack which fuels its desire? Hence, if, as aforementioned, the brain, as an index of the real, lacks both a body and a psyche, then it can be argued that it is from here that the brain image receives its interpellative power. The brain is presented to us as a *simulacrum*, which, in Baudrillard's conceptualization of the term, is neither a symbol nor a reproduction of something else: it is, rather, the ersatz of something that was always missing, something which is (necessarily) lacking. Therefore, it is precisely as the semblance of an ultimate Real that the brain image is capable of capturing our fixation and transfixing us^17^.

But, in the interest of clarity, it is important to stress that the brain is a simulacrum precisely because it is the ersatz of the *absent* psyche. For, it can be argued that the modern psyche represents nothing but a signal of the human being having lost any of its ontological ground since the advent of modernity. In fact, the emergence of modern psychology during the Enlightenment corresponded with an epochal problematization of subjectivity itself. In one respect, the subject was at risk of being engulfed by the massive objectifying potential of the sciences, while in another respect, it was only science that could offer it the necessary reference or anchoring points hitherto provided by God and religious discourse^18^. The psyche, ultimately, stands for the very ontological abyss laid bare by modernity.

One way in which this notion of the brain image as the transfixing simulacrum of psychology can be apprehended, is via Adorno's, McLuhan's and Baudrilllard's conception of late-modern media as being characterized by a predominance of form over content (Taylor, 2008). We appear to be fascinated by the form, the sulci, the gyri, the deeper structures, etc., and are not so concerned by the fact that this form can only be filled with the rather meager (non)content of psychology (which reduces us to individuals with a limited if not standardized behavioral, cognitive, and emotional life). If the neurosciences can be said to be reductionist, then this is because the reduction has already been done at a prior stage. Psychology is always already, and indeed inevitably, in the business of trading content for forms (empty forms barely concealing the structural absence of content), which are ideally suitable for subsequent mapping onto the brain. Even if this mapping of psychology to the brain is done in a sophisticated fashion (trading neo-phrenology with a dynamic network approach and/or attributing plasticity to brain-processes, for example) the neurosciences nevertheless always remain susceptible to regressing back within a correlationist-reductionist scheme.

The issue of the brain requiring filling in by psychology resonates strongly with the fact that, via the same virtual and image technologies used in brain imaging, our life, relationships, and work increasingly take place on virtual platforms such as Facebook, Twitter, Instagram: our presence on these social media sites is also fleshed out with psychology. We like or no longer like, we have friends and networks, we have a life-line, we have events. In other words, social media technology prompts us to act like decent subjects: in fact, we are literally stuffed to the brim with psychology. Do you like this? Why do you not like this? Do you want to share it, put it on your lifeline? Remember, it's your mother's birthday in a few days… Our virtual double cannot remain empty, it has to be fleshed out in psychological terms, our virtual avatar needs to be dressed, very specifically, in psychological robes.

This filling up of the empty virtual space with psychology, not surprisingly, is precisely at hand in the rationale of the aforementioned HBP:

Neuroscience and medicine both require an integrated multi-level understanding of brain function in the context of cognition and behavior. The HBP ICT platforms would provide a new foundation for this kind of research. Once brain models have been integrated with a simulated body acting in a simulated environment and trained to display a particular competency, neuroscientists would be able to systematically dissect the neuronal mechanisms responsible, making systematic manipulations and measurements that would be impossible in the lab (Walker, 2012).

Hence, returning to the desire of the brain image, do we not find ourselves in precisely the same situation as the story Pinocchio? That is, the imaged and virtual brain desires to become human, it wants to be fleshed out with psychology. Neuroscience needs psychology, mainstream cognition, and behavior psychology, to construct the architecture of its scene and to position its avatars within this scene.

But, of course, the significant aspect of all this is that the HBP is not that far removed from everyday life: indeed, if the program intends to study simulated bodies in simulated environments, is this not what everyday life is actually like in contemporary societies? After all, are not the “variables” under research “perception and action, decision-making, goal oriented behavior, navigation, multisensory perception, object recognition, body perception… ” (Walker, 2012) not precisely the same variables that structure our actual lives? That is, whether in education, parenting, schooling, work, etc., it is these items that we are continually told matter, these items that both structure what is done (and what we ourselves do) and form the basis on which we are evaluated. Again, everybody here is hailed into adopting the external point of view: the toddler knows what empathy is and why it is important, the parent knows the theoretical background of positive reinforcement, and the manager instructs his workers into the brain-based psychology of being goal-oriented.

So, again, the question arises: should we concentrate our critical efforts upon finding ways to protect the human from these forms of (neuro)psychologisation, visualization, and virtualization? To stand firm and assert that the human (or the revolution) will not be fMRI-zed? However, the conclusion of this paper is that what should concern us, in actual fact, is not so much the possible mismatch between the human (as he or she would allegedly really be) and the virtual, but, rather, the potential gap between the imagined, analog-psychological human, and the virtual, digital-neuronal human. For, as I've hopefully made clear, any attempt to defend a supposedly real or true human would inevitably only lead to recourse to another version of psychology or meta-metapsychology, which, in turn, would unwittingly lead to yet another virtualization. To look for a subject beyond the image and the psychologized imagery is thus a dead-end.

Resultantly, it is perhaps expedient to follow Mitchell's suggestion that there is something in the image itself which resists digitalization (Mitchell and Smith, 2008). Mitchell argues, on the basis of a series of paintings including René Magritte's famous “Ceci n'est pas une pipe”-painting (picturing a pipe together with the caption “this is not a pipe”), that the picture “offers a presence and insists on an absence in the same gesture” (Mitchell and Smith, 2008). Is this not precisely what the brain scan does also? It appears to offer a massive, ontological and fully fleshed out human being, whilst simultaneously serving as the personification of the notion that there is nobody at home in the brain as such: in fact, if anything, the multi-colored charts signal that the ghost in the brain has fled. But Mitchell also adds that, beyond this dynamic of presence and absence, images also testify to an excess, an additional density or plenitude, a kind of “surplus” of presence (Mitchell and Smith, 2008). In the brain image this surplus manifests itself in a very specific sense: the brain knows, feels and experiences more than we think (or know, feel, and experience). Subconsciously, allegedly, archaic emotions are in play, computations are done and cognition is mobilized all without us having the faintest clue of what is happening (see e.g., Ledoux, 1996; Libet, 1999). And it is here where the ghost in the brain seems to reappear, albeit as a sort of homunculus inhabiting our skull. The psychology allocated to the brain in the end cannot be ours, it is the brain's. In this way, one could say that the function of today's neuropsy-discourses is above all to contain and to tame this extra brain-man gone wild, this psychological *Übermensch* living inside our head. Mitchell himself perspicaciously tries to see in the “pictorial turn” in the twenty-first century a “biopictorial turn,” pointing to “the production of copies, simulations, or reproductions of living organisms and organs, and along with this, a resurgence of ancient fears about “doubles,” evil twins, and the loss of identity” (Mitchell and Smith, 2008). Concerning these “biocybernetics,” Mitchell contends that the enigma and the defiance for our understanding is that, while, on the one hand, image culture is today fully digitalized, on the other hand, there is something of the dimension of the analog sticking in the image itself:

[O]n the one hand, we live in the ‘digital age’, and, on the other, […] images – analog signs, mind you – have now taken on a new and unprecedented power. We will not be able to keep our bearings in the new visual and mediatized worlds that are opening before us unless we grasp firmly at both horns of this dilemma (Mitchell and Smith, 2008).

Indeed, even if imaging today has become fully digital—and hence only now the unbound reproductive potential of the image (and hence its indestructibility) is fully unleashed—the image remains, at its functional, phenomenological level, analog. This is why a picture, given that its digital format in the end does not coincide with the image itself (the Gestalt-like form)^19^, is not “googleable” as such. To illustrate this fact: I once found a (rather iconic) image on a website, albeit without the proper citation information: subsequently, I wanted to reuse the image in a paper and thus required a reference regarding the source of the image. However, to my annoyance, I have to admit, I found myself faced with the impossibility of googling it directly, so I had to take recourse to discursive descriptions of the image. However, in the interim Google has developed “Google Image Search,” which allows you to search for other images by uploading or dragging an image into the search box itself. This clearly represents an attempt to try to get a grip on the analog character of the image, as the search is based on captions, meta-tags, data found on the webpage where the image is located, and to a certain extent image recognition (using e.g., color patterns). Experimenting with some self-made and on the spot generated images (which obviously have no discursive tags or connections whatsoever), does generate some results which show vaguely similar images (color patterns do seem to be the main criterion), however, ultimately, this testifies to the tenacity and the resistance of the *analogness* of the image.

It would appear, then, that we have to conclude that Logos (destined to go virtual and digital) does in fact have its remainder, its excess, and that this is precisely the image qua analog image. The brain image is, ultimately, not digital, but analog. As aforementioned, in order to shift from brain data to brain images the human gaze must be brought in: the human factor, in other words, is the image. The movie *The Matrix* illustrates this point nicely: in certain scenes the virtual life-world of the avatars becomes visible in its true form, that is, as green data (the so-called “digital rain”), scrolling down over the screen^20^. The central idea of the movie is that in order for the digital to come alive, that is, to become image, the human factor is required. Again, the human factor is the image, which, on the one hand, makes digitalization and virtualization possible, whilst, on the other hand, resisting full digitalization by introducing the analog into the virtual. If in pre-modern times image culture was tributary to the Christianization of Roman iconography (see Buck-Morss, 2007), today the two central forces that define our present-day virtual image culture are science and the corporate media. Any viable resistance and critique will have to start from the fact that, in this digital day and age, the medieval dictum “The primordial rose abides only in its name; we hold names stripped” no longer holds, today it is the stripped images that we hold on to.

Let me make reference to an artistic attempt to do so: the Flemish artist Jan Fabre's work “Madonna.” In a remarkable version of what for Buck-Morss is the icon par excellence, the Madonna with child (*Theotokos*, the point between divinity and humanity), Fabre's pieta portrays the artist himself as the figure of the dead Christ. While Fabre depicts Maria's face as a skull—the skull of early modernity, reminiscent of the pre-modern *memento mori* and the modern drawings of Vesalius—the artist, as the son of god, depicts what we could understand as the late-modern equivalent of the skull, that is, the brain. However, remarkably, the brain is not positioned inside his head, but, instead, is almost casually held in the right hand of the son-figure. Such a gesture fully elevates the brain into the position of an icon, in the sense that it is not coextensive with the subject itself, but, rather, its excess. At the most, one could say it is the point of liaison between the subject and the Human as such. The brain appears precariously close to dropping out of the hand of the figure of the artist, and falling onto the ground. This could point to the vulnerability of the human, e.g., the vulnerable brain, but it could also be drawing attention to the fact that the brain, having become an icon, is inevitably always on the verge of slipping out of one's hand. Each and every icon, it could be said, begs for iconoclasm whilst simultaneously resisting it, by virtue of it being analog. Therefore, perhaps, in the whole installation in Vienna, the majestic pieta is surrounded by four giant (if not obscene) cerebellums, each of which is adorned with a religious attribute or artistic (auto) reference. One might be forgiven here for interpreting the multiplication of these massive, heavy, analog brains in a Freudian way: the proliferation of the brains signals the absence of the psyche.

## Conclusion

If today we increasingly live in a visual culture whereby the primary way in which we relate to ourselves and the world is via the image, then it is fair to say that this image is constructed and validated by the techno-scientific discourses. In this paper I have tried to trace the pathways of this complex scheme. Firstly, I have argued, by virtue of drawing upon but eventually transcending an Althusserian interpellative framework, that the (late)modern construction of subjectivity is invariably centered around the adoption of an academic point of view. This is an important point, I claim, if one wants to understand how neuroscience is in danger of being unknowingly drafted into contemporary bio-politics: the identification with the alleged a-political vantage point of Academia is the precise form of contemporary ideological interpellation.

Secondly, I have demonstrated that brain imaging cannot be cut loose from the far-reaching digitalization and virtualization of our life-world. To summarize my conclusions on this point in an admittedly bold statement: if left unquestioned, this close link threatens to invalidate the whole project of brain imaging.

Thirdly, I have considered and, ultimately, rejected the possibility that psychology, given that it presumably would resist the force of neuro-colonization, could provide the basis on which to understand the conditions and boundaries of neuro-imaging. As it were, psychology, rather than being the solution or a locus of resistance, is actually the unacknowledged and structurally unsolvable problem of the brain sciences.

Fourthly, I have closely examined the ways in which the iconographic brain is constituted via what I have called the data-gaze. I have posited that, at the site of the production of academic knowledge—i.e., at the site of the object and how this is constructed by science—the latter inevitably harks back to the subject and its intricate and paradoxical auto-construction (the subject as both constituted and constituting). Brain data is hence always already visual data, as they derive from an encoding of the subjective gaze within the activity of data collection itself. And, moreover, it is in relation to this paradoxical objectifying/subjective gaze that the so-called lay-man identifies him or herself.

Fifthly, I have argued, through recourse to Mitchell's reconceptualization of iconology, that the fundamental question to ask is: what does the brain image want from us? This is where a deconstruction, or rather a decentering of psychology can be effected: what the brain image lacks is a body and a psyche, and it's only from this vantage-point that its powerful interpellative force becomes clear. This is because it is precisely there that the neurosciences get caught within processes of psychologisation and virtualization, and, in turn, risk regressing back within a correlationist-reductionist scheme. At that point, I concluded that, given both the hegemony of the neuroscientific virtualized image culture and the fact that attempts to look for a subject beyond the image has proved to be a dead-end, the only viable basis for a critical approach is to be found in the analog-ness of the image itself.

By way of reiterating this albeit somewhat paradoxical conclusion, and in an attempt to show its bearings, I would argue that we owe the instantiation of the Academic vantage point, a vantage point that this paper has demonstrated is central to the operation of brain image culture, to René Descartes' *epoché*, his contemplative retreat from the world in order to be “more a spectator than an actor in all the comedies that are played out there” (Descartes, 1996[1637]). Descartes' positioning of himself in the theatre seat so to speak constitutes the point of departure for the modern objectifying gaze, which, in turn, creates the imagery and the scene that is looked upon. Continuing the dramaturgical metaphor, it is immediately apparent that the visual culture we live in needs scenario's and scripts in order to structure and organize both what is seen and what happens within the scene. For Descartes, this was his “provisory moral”: searching for truth and suspending all certainties, he adopted a provisory code of morals in order to be able to locate himself in the world. For the late-modern subject, I would argue, this is the specific function carried out by the neuropsy-sciences, in the sense that, via the medium of the brain images, they flesh out the scripts that structure what we perceive and how we navigate our way through the scene (De Vos, 2013a). It is only through full acknowledgment of the fact that any analysis of this scheme will invariably culminate in the irreducibility of both the gaze and the image (and its stubborn analog-ness) that any critical position can arise from where to assess the reductive and bio-political entanglements of that scheme.

### Conflict of interest statement

The author declares that the research was conducted in the absence of any commercial or financial relationships that could be construed as a potential conflict of interest.

## Footnotes

^1^Of course, as Rose and Abi-Rached write, most neuroscientists are careful to speak of neural correlates of mental processes, but “[t]heir press releases and media accounts are less cautious” (Rose and Abi-Rached, 2013).

^2^The specific notions of critique and resistance will be explained in that section.

^3^See also the Dutch best-seller by brain researcher Swaab (2011).

^4^“The stepping out of (what we experience as) ideology is the very form of our enslavement to it” (ŽiŽek, 1994).

^5^See e.g., Kurzweil (2005), for a discussion see Chalmers (2010), and for a critique Nicolelis (2013).

^6^As stated on: https://www.humanbrainproject.eu/nl_BE/discover/the-project/research-areas

^7^https://www.humanbrainproject.eu/nl_BE/discover/the-project/research-areas; https://www.youtube.com/watch?v=_UFOSHZ22q4

^8^For an assessment of these kinds of resistance against neuroimaging see, for example, Whiteley (2012).

^9^See for example the website “Visual recognition,” a spin-off of the ISLA laboratory of the University of Amsterdam: http://www.visual-recognition.nl/. Although, of course, this could very easily be criticized on the basis of it being an overly artificial assessment of emotions, in which they are divided into a limited array of fixed categories.

^10^See Eugene Thacker's claim that biology is always already digital (Thacker, 2004).

^11^http://www.med.harvard.edu/AANLIB/cases/caseNA/pb9.htm

^12^http://www.amenclinics.com/the-science/spect-gallery

^13^e.g., the BRAIN initiative (Brain Research through Advancing Innovative Neurotechnologies) http://www.youtube.com/watch?v=uJuxLDRsSQc.

^14^http://www.humanconnectomeproject.org/gallery/

^15^http://news.bbc.co.uk/today/hi/today/newsid_8241000/8241440.stm

^16^Buck-Morss argues that while, on the one hand, Christianity took over much of Roman iconography, on the other hand, once the Roman Empire was Christianized, the connection allowed a transcendent claim for sovereignty (Buck-Morss, 2007).

^17^Or, as Baudrillard observes: Any system that is totally complicit in its own absorption such that signs no longer make sense, will exercise a remarkable power of fascination (Baudrillard, 1990).

^18^This Catch 22 situation can be reformulated as follows: both the modern psyche and modern psychology are Enlightenment's stillborn children (De Vos, 2013a).

^19^Also Mitchell stresses the need to distinguish between the image and the picture (Mitchell, 2005).

^20^Of course this is still image based language.
